# Muscle-Type Specific Autophosphorylation of CaMKII Isoforms after Paced Contractions

**DOI:** 10.1155/2014/943806

**Published:** 2014-06-26

**Authors:** Wouter Eilers, Wouter Gevers, Daniëlle van Overbeek, Arnold de Haan, Richard T. Jaspers, Peter A. Hilbers, Natal van Riel, Martin Flück

**Affiliations:** ^1^School of HealthCare Science, Institute for Biomedical Research into Human Movement and Health, Manchester Metropolitan University, Manchester M1 5GD, UK; ^2^Laboratory for Myology, Faculty of Human Movement Sciences, MOVE Research Institute Amsterdam, VU University Amsterdam, 1081 BT Amsterdam, The Netherlands; ^3^Department of Biomedical Engineering, Eindhoven University of Technology, 5612 AZ Eindhoven, The Netherlands; ^4^Laboratory for Muscle Plasticity, Department of Orthopedics, University of Zurich, Balgrist University Hospital, Forchstrasse 340, 8008 Zurich, Switzerland

## Abstract

We explored to what extent isoforms of the regulator of excitation-contraction and excitation-transcription coupling, calcium/calmodulin protein kinase II (CaMKII) contribute to the specificity of myocellular calcium sensing between muscle types and whether concentration transients in its autophosphorylation can be simulated. CaMKII autophosphorylation at Thr287 was assessed in three muscle compartments of the rat after slow or fast motor unit-type stimulation and was compared against a computational model (CaMuZclE) coupling myocellular calcium dynamics with CaMKII Thr287 phosphorylation. Qualitative differences existed between fast- (*gastrocnemius medialis*) and slow-type muscle *(soleus)* for the expression pattern of CaMKII isoforms. Phospho-Thr287 content of *δ*A CaMKII, associated with nuclear functions, demonstrated a transient and compartment-specific increase after excitation, which contrasted to the delayed autophosphorylation of the sarcoplasmic reticulum-associated *β*M CaMKII. In soleus muscle, excitation-induced *δ*A CaMKII autophosphorylation demonstrated frequency dependence (*P* = 0.02). In the glycolytic compartment of *gastrocnemius medialis*, CaMKII autophosphorylation after excitation was blunted. *In silico* assessment emphasized the importance of mitochondrial calcium buffer capacity for excitation-induced CaMKII autophosphorylation but did not predict its isoform specificity. The findings expose that CaMKII autophosphorylation with paced contractions is regulated in an isoform and muscle type-specific fashion and highlight properties emerging for phenotype-specific regulation of CaMKII.

## 1. Introduction

Myocellular calcium is an important second messenger of muscle regulation. This role is mediated by increases in sarcoplasmic calcium concentration with functional recruitment of muscle fibers following motoneuron-induced muscle excitation (i.e., recruitment; [1]). The elevated calcium initiates sarcomere shortening, that is, excitation-contraction coupling, and produces downstream effects on gene regulation, that is, excitation-transcription coupling [2–5]. Three types of motor units can be distinguished based on their contractile and metabolic characteristics, that is: a fast-fatigable, a fast-fatigue-resistant, and a slow-fatigue-resistant type [1, 6]. Thereby it is observed that slow and fast contractile characteristics of motor units are reflected by differences in the (electric) firing pattern of the innervating motoneuron and concentration differences in the rise of myocellular calcium with excitation [7]. Experimental studies have established a specific preference of motoneuron activation frequencies for the three contractile types of motor units [1, 8]. Slow fatigue-resistant motor units are preferentially recruited by low-frequency depolarisation of motor units. In contrast, recruitment of fast-type motor units often occurs at higher stimulation frequencies [9, 10]. Furthermore, the elevated content of the organelles of excitation-contraction coupling, t-tubuli and sarcoplasmic reticulum, in fast-type fibres is associated with larger increases in myocellular calcium concentration during recruitment [11].

Along with the calcium/calmodulin-dependent phosphatase calcineurin [7, 14] changes in the activity of calcium/calmodulin dependent kinase II (CaMKII) are a potential interface explaining the downstream effects of elevated myocellular calcium on calcium channel activity and gene regulation [7, 12, 14] and indicated differences between muscle fiber types [7]. To determine the relevance of CaMKII for the regulation of calcium channel activity and gene expression, it is important to understand the regulation of CaMKII activity. CaMKII is a multimeric phosphotransfer enzyme that assembles from different isoforms [15]. Its phosphotransfer activity is induced following rises in intracellular calcium after autophosphorylation of Thr287 in its autoinhibitory domain, enhancing its phosphotransfer activity and rendering it calcium-independent through a subsequent conformational change [15]. Thr287 phosphorylation of CaMKII and a concomitant increase in calcium/calmodulin-independent phosphotransfer activity can be observed within seconds after onset of muscle contraction [12]. Based on model studies of cardiac and brain isoforms, this increase in CaMKII autophosphorylation is believed to rapidly fall within seconds to baseline levels [16, 17]. This is, however, not what can be observed in skeletal muscle where autonomous CaMKII activity can remain specifically elevated after prolonged repetition of contraction, such as running type exercise [18].

Four isoforms of CaMKII (i.e., *β*M, *δ*A, *δ*D, and *γ*B) have been reported to exist in skeletal muscle [12, 13].* In vitro* measurements point out characteristic differences in calcium sensitivity of activation between CaMKII isoforms [19, 20]. The biochemical observations are in line with cellular investigations that allocate different functions for the various CaMKII isoforms. For instance, the *β*M isoform has been found to associate with sarcoplasmic reticulum hinting that it possibly operates in the regulation of calcium channels involved in muscle contraction and relaxation [13]. Conversely, the *δ*D/*γ*B CaMKII, and possibly *δ*A, isoforms are associated with nuclear functions [21–23].* In vitro* studies of CaMKII revealed that CaMKII autophosphorylation is subject to discrete regulation by the frequency and amplitude of calcium transients, which differs between CaMKII isoforms [24]. Studies in hippocampal cultures identified that Thr286-phosphorylation of the neuron-specific CaMKII isoform *α* acts as a frequency/number decoder for sensory input [25]. Computational modeling of CaMKII activation by postsynaptic calcium pointed out that autophosphorylation of *β* CaMKII demonstrates a greater response range than *α* CaMKII [26]. The extent to which isoform-specific CaMKII autophosphorylation is implicated in the response of muscle fiber types to functional recruitment is not understood.

Towards this end we characterised the pattern of CaMKII isoform expression and CaMKII autophosphorylation following electrically paced contractions in phenotypically distinct motor compartments of the rat using muscle-tendon preparation* in situ*. Specifically we asked whether autophosphorylation of the different isoforms of CaMKII is subject to the same regulation by motoneuron excitation and whether this would differ between three distinct compartments of the* triceps surae* muscle group, representing the three types of motor units, when excitation is paced with slow-type and fast-type electric stimulation protocols [9, 10]. Emphasis was put on the question of whether the reported contribution of mitochondria to calcium buffer capacity would exert an influence on Thr287 content of CaMKII [27]. We then asked whether we can reveal mechanistic understanding of the properties of excitation-induced CaMKII regulation between muscle types and isoforms by confronting experimentally observed “on-” and “off-rates” of CaMKII Thr287-phosphorylation with results from a newly assembled computational model.

## 2. Materials and Methods

### 2.1. Design

Four experimental protocols were run to pace contraction of the* triceps surae* muscle group of one leg of rats with a slow and fast motor unit-targeted protocol [8, 9]. Three muscle compartments of the* triceps surae* were rapidly isolated and subjected to the measure of CaMKII Thr287 phosphorylation with biochemical means. The nonstimulated muscles of the contralateral side served as controls for Protocols 1 and 2. Collaterally, CaMKII Thr287 phosphorylation was assessed using an* in silico* model combining myocellular calcium dynamics with CaMKII isoform autophosphorylation in different muscle types.

### 2.2. Animals

Three-month-old female Wistar rats (Harlan Laboratories) were anaesthetized by intraperitoneal injection of 1.2 mL/100 gram body weight of 12.5% urethane. Ear and foot reflexes were tested to check whether the animal was sufficiently anaesthetized. Subsequently, injections of 0.3–0.5 mL, up to a maximum of 1.5 mL, were given every 10 minutes afterwards until reflexes had disappeared. Rats were kept on a heated pad (37 ± 0.5°C) to prevent hypothermia. Experiments were carried out with approval of the local Animal Experiments Committee at the MOVE Research Institute Amsterdam, VU University Amsterdam. 12 and 20 animals entered the experimental Protocols 1 and 2 (mean body weight of 191–230 grams) and Protocols 3 and 4 (mean body weight of 205–220 grams), respectively.

### 2.3. Muscle-Tendon Preparation

Hind limbs were shaved and the skin was removed, after which* gastrocnemius medialis *and* soleus *muscles were exposed and mechanically isolated by removing as much as possible the myofascial connections to surrounding muscles. Blood supply to and nerve innervations of* m. gastrocnemius medialis *and* m. soleus *were kept intact. The calcaneus was cut from the talus, while still attached to the Achilles tendon. The sciatic nerve was dissected free, proximally severed, and electrically stimulated through an electrode by different protocols being controlled by a computer.

**Protocol 1 (150 Hz protocol).**

*Gastrocnemius medialis *and* soleus* muscles of the right leg were kept below slack length (without determination of optimal length) and electrically stimulated via the sciatic nerve with a train of 100 rectangular electric pulses of 50 microseconds duration and 3 mA amplitude delivered at 150 Hz. Muscles of the left leg served as nonstimulated controls. The experiment was performed at a room temperature of 23°C.

**Protocol 2 (10 Hz protocol).**

*Gastrocnemius medialis* and* soleus* muscles were electrically stimulated as described for Protocol 1 but with the modification of a single train of 100 pulses at 10 Hz.

**Protocol 3 (tetanic contraction at optimal length).**

3 tetanic contractions with 2 minutes of rest in between were imposed on* gastrocnemius medialis* muscle via the sciatic nerve with a modification of the 150 Hz-protocol where the train consisted of 30, rather than 100, electric pulses of 50 microseconds duration and 3 mA amplitude, at 150 Hz. The temperature of the* gastrocnemius* muscle was kept at 35°C using an envelope into which warm water vapor was sprayed.

**Protocol 4 (24 tetanic contractions).**

A 2 minute protocol of 24 tetanic contractions (1 contraction every 5 seconds) with the same duration and frequency as described for Protocol 3 was imposed. The experiment was performed at 35°C. This protocol resulted in a decrease in maximal tetanic force of approximately 30%.

### 2.4. Sampling

After completion of the protocol, the proximal (i.e., oxidative) and distal (i.e., glycolytic) portion of* gastrocnemius medialis* muscle [28] and* soleus *muscle (in Protocols 1 and 2 only) was dissected as rapidly as possible and snap-frozen in liquid nitrogen. The first muscle was typically sampled after 1 minute and the second after 2 minutes. In half of the experiments,* soleus* muscle was sampled first, and in the other half* gastrocnemius medialis *muscle was sampled first. Subsequently, the nonstimulated left muscles were dissected and snap-frozen. Muscles subjected to Protocol 4 were subjected to sampling over a time-course covering 10 and 60 minutes after contraction to estimate the rate of CaMKII dephosphorylation. Rats were subsequently euthanized by intracardial injection of Euthasol while fully anaesthetized. Muscles were stored at −80°C until used for western blot analysis.

### 2.5. Biochemical Analysis of CaMKII Thr287 Phosphorylation

25 *μ*m thick cryosections were prepared from the frozen muscles, pooled and homogenized on ice using a Polytron homogeniser (Kinematica AG, Luzern, Switzerland) in ice-cold RIPA buffer (50 mM TRIS-HCl (pH 7.5), 150 mM NaCl, 1 mM EDTA, 1% v/v Nonidet P40 substitute, and 0.25% w/v sodium deoxycholate, including freshly added protease/phosphatase inhibitors: 1 mM NaF, 1 mM Na_3_VO_4_, 0.1 mM PMSF, 1 *μ*g/mL leupeptin, 0.2 *μ*g/mL pepstatin, and 0.1 *μ*g/mL aprotinin. Chemicals were obtained from Sigma-Aldrich (Poole, United Kingdom) unless stated otherwise. Crude homogenates were aspirated 5–10-times through a 0.8 mm syringe needle and stored at −80°C until being further processed. An aliquot of the aspirated homogenate was taken for determination of protein concentration with the bicinchoninic acid protein assay (Pierce, Rockford, IL, USA).

Protein levels of total CaMKII, phospho-Thr287-CaMKII, and cytochrome-c oxidase subunit IV (COXIV) were analyzed by western blotting followed by immunodetection. Homogenates were denatured by addition of sample buffer (final concentration: 50 mM TRIS-HCl (pH 6.8), 2% w/v SDS, 10% v/v glycerol, and 2% *β*-mercaptoethanol) and 5 minutes heating at 95°C. 20 *μ*g of protein per lane was separated with SDS-PAGE on a 15% acrylamide (Biorad) gel and transferred overnight in ice-cold buffer onto a nitrocellulose membrane (GE Healthcare Life Sciences, Little Chalfont, United Kingdom). Membranes were stained with Ponceau S solution to confirm equal protein loading and transfer and then subjected to immunodetection. After blocking, 2-hour incubations were carried out with primary antibody against pan-CaMKII (BD Bioscience, no. 611292, dilution: 1/2500), phospho-Thr287-CaMKII (Cell Signalling Technology, no. 3361, dilution: 1/1000), or COXIV (Cell Signalling Technology, no. 4850, dilution 1/2000) in TTBS (20 mM TRIS-HCl (pH 7.5), 0.9% w/v NaCl, 0.05% Tween-20) with 5% milk (pan-CaMKII, COXIV) or 5% bovine serum albumin (phospho-Thr287-CaMKII) as blocking agent. After serial washes in TTBS, membranes were incubated with species-specific horseradish peroxidase-conjugated secondary antibodies and signal detected enhanced chemiluminescence (Pierce, Rockford, IL, USA) and recorded with a ChemiDoc XRS system (Biorad, Hemel Hempstead, United Kingdom).

Samples from the oxidative and glycolytic* m. gastrocnemius medialis *and* m. soleus *were analyzed on separate immunoblots. For each muscle compartment, two sample pairs of stimulated and nonstimulated contralateral control muscles from Protocols 1 and 2, respectively, were analyzed on a same blot. Signal intensity of the protein band of interest was quantified using Quantity One version 4.6.8 (Biorad), background-corrected, and normalized to the average of CaMKII proteins signals from all lanes on the blot. Then for each blot, the normalized signals were related to the total CaMKII signal for the respective resting (i.e., “nonstimulated”) muscle and data pooled between experiments. Finally the values were related to the mean of the “nonstimulated” samples for the respective experimental protocol and subjected to statistical analysis.

The specificity of CaMKII and phospho-Thr287-CaMKII detection was ensured in control experiments monitoring calcium/calmodulin-inducible phosphorylation in “cold”* in vitro *kinase assays as described [18]. In brief, 5 *μ*L of total muscle homogenate was suspended in a reaction mix containing calcium/calmodulin (Enzo Life Sciences, Exeter, United Kingdom) (total volume: 50 *μ*L; 0.1 mM ATP, 10 mM HEPES (pH 7.4), 5 mM MgCl_2_, 0.1% Tween-20, and 0.5 mM CaCl_2_ + 1 *μ*M calmodulin or 5 mM EGTA) for 30 minutes at 30°C. The reaction was stopped by the addition of 16.7 *μ*L 4x sample buffer. Samples were heated to 95°C and proteins were separated with SDS-PAGE on a 7.5% (or 15%) acrylamide (Biorad) gel and subjected to western blotting as described. Based on data from [13], we identified the detected CaMKII bands as *β*M (72 kDa), *δ*A (60 kDa), and a combination of *δ*D and *γ*B (58 kDa), the latter two of which could not be separated in all gels. Therefore, these two CaMKII isoforms were assessed as one band.

### 2.6. Model Development

We set out to develop a computational model to predict calcium driven changes in Thr 287 phosphorylation of CaMKII (termed CaMuZclE for CaMKII muscle model from Zurich-Eindhoven). The model is based on a spatiotemporal model of calcium dynamics in the half sarcomere of fast-twitch muscle [29] and a biochemical model describing CaMKII activation [16] which was reduced to a lumped version by removing the spatial component.

**Figure 1:**
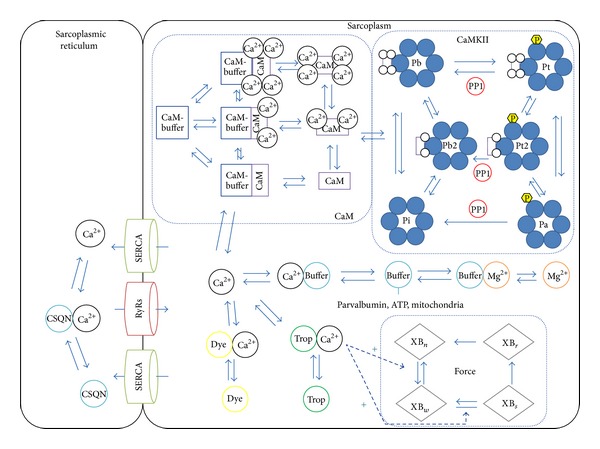
Schematic representation of the components of the CaMuZclE model to simulate calcium-driven Thr287 phosphorylation of CaMKII in skeletal muscle. The model includes calcium flow through the sarcoplasmic reticulum and sarcoplasm. Calcium is bound to the buffer calsequestrin in the SR and is transported by RyRs channel and SERCA pump between the SR and sarcoplasm. Within the sarcoplasm calcium binds to the buffers ATP and parvalbumin, mitochondria, and troponin C, initiating sarcomere shortening and force production. Furthermore, calcium binds to the calcium sensor calmodulin, which in turn activates the phosphotransferase CaMKII, initiating several pathways. A comprehensive list of all abbreviations can be found in the Appendices.

The model is based on the interactions between the chemical species inside a sarcomere, consisting of the ions calcium, magnesium and potassium, the calcium buffers calsequestrin, parvalbumin, troponin, adenosine triphosphate (ATP), and mitochondria that act as a calcium buffer, the proteins calmodulin (CaM) and calcium/calmodulin-dependent kinase (CaMKII), CaM buffers, and a calcium-binding dye. Both the sarcoplasmic reticulum and the sarcoplasm are modelled, including the pumps facilitating transport of calcium between the compartments, Ryanodine receptors (RyR), and sarco/endoplasmic reticulum calcium-ATPase pumps (SERCA). Furthermore, the development of contractile force created by contraction is modelled. Reactions are described in terms of changes in the concentration (fluxes) of respective species using coupled ordinary differential equations (ODEs) based on a set of initial concentrations and parameters of the reaction rates. The system of ODEs resulting from these sets is then solved using a numerical integration algorithm, in this case the Matlab (The Mathworks) built-in solver ode15s using an implicit integration scheme with numerical differentiation formulas. The calculation takes around 20 seconds to simulate 10 minutes of experimental time using an Intel core i7 processor (3.4 GHz). Memory usage is negligible (<100 MB). The model was calibrated by measures on sarcoplasmic calcium based on the calcium-binding dye. A schematic representation of all the model components and fluxes can be seen in Figure 1. Sensitivity analysis was performed using the multiparametric sensitivity analysis (MPSA) method, describing the speed of the calcium pumps, total calsequestrin concentration, and total CaMKII concentration as most influential parameters (Table 1). Further details on the model equations can be found in the Appendices and Tables 2–6. MPSA was conducted by uniform sampling of 4000 parameter sets from an interval from 75% to 125% of the default parameter set using Latin hypercube sampling, ensuring the sets cover the complete parameter space. Stimulation with a pulse train of 100 pulses at 150 Hz was used (Protocol 1), as this describes a full and complete stimulation. For each parameter set the time course of phospho287-CaMKII was compared to the output with the default set. A sum of squared differences was used as measure. Subsequently, sensitivity values were calculated as described [30].

Interdependent parameters were excluded as these variables change together with the variables they are dependent on. Exponentials of the description of the RyR activity were excluded as well as the RyR activity is regulated by varying the parameter CaMax. The remaining parameters are ordered with respect to their influence on the trace of phosphorylated CaMKII, as calculated via the MPSA method. The MPSA results were used to perform uncertainty analysis [31]. Poisson distributions were fitted to the set of output transients resulting from MPSA and were used to draw 95% confidence intervals. For clarity, only the trace of the *β*_M_ CaMKII isoform is presented; the traces of the other isoforms display similar behavior.

### 2.7. Statistics

All statistical tests were performed using Statistica 10.0 (Statsoft Inc., Tulsa, OK, USA). The effect of stimulation with Protocols 1 and 2 on total and phospho-Thr287-CaMKII levels was tested with repeated-measures ANOVA on the factor “stimulation” [stimulated, rest] with a post hoc test of Fisher. Additional factors were “CaMKII isoform” [*β*M, *δ*A, *δ*D/*γ*B], ‘stimulation frequency' [10 Hz, 150 Hz], and muscle compartment [glycolytic* gastrocnemius medialis*, oxidative* gastrocnemius medialis*, and* soleus*]. These were run separately or in combination. The effect of stimulation with Protocols 3 and 4 on phospho-Thr287-CaMKII levels was tested with ANOVAs on the factor “stimulation” [rest, after 3 tetanic contractions, after 24 tetanic contraction] × “muscle compartment” [glycolytic* gastrocnemius medialis*, oxidative* gastrocnemius medialis*] × “CaMKII isoform” [*β*M, *δ*A, *δ*d/*γ*B] with a post hoc test of Fisher. Two-sided post hoc tests were carried out to analyze the effect of electric stimulation on phospho-Thr287-CaMKII.

## 3. Results

### 3.1. CaMKII Isoforms in Skeletal Muscle

**Figure 2:**
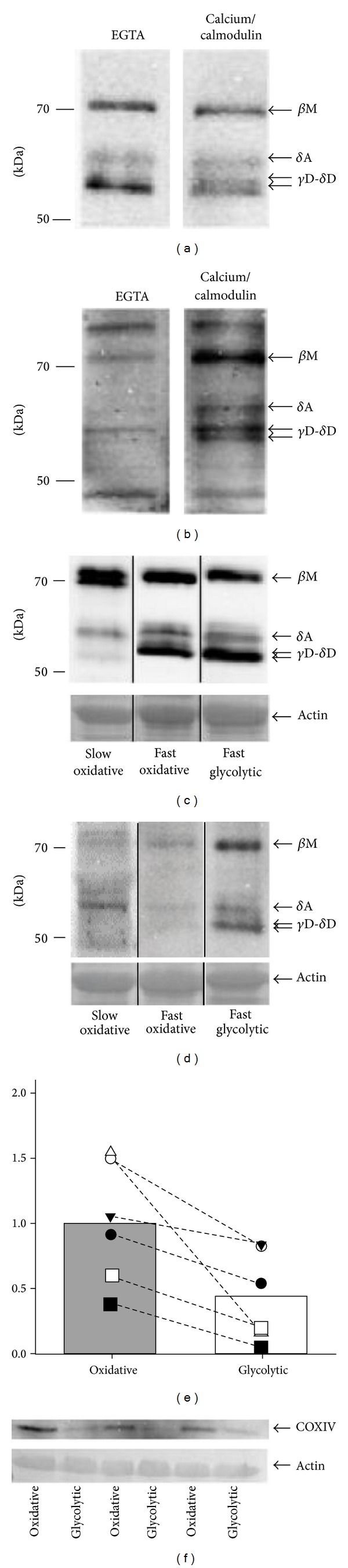
Calcium/calmodulin-dependent kinase II isoforms in rat skeletal muscle. Total homogenate of a* gastrocnemius* muscle from a rat was prepared and subjected to* in vitro* kinase reactions in the presence of EGTA or calcium/calmodulin and was subjected to immunoblotting with a pan-CaMKII (a) and phospho-Thr287 specific antibody (b). CaMKII isoforms were then assigned based on a calcium/calmodulin-inducible phospho-Thr287 signal and a detection of similar sized bands according to the nomenclature established by Rose [12] and Bayer [13]. (c)(d) CaMKII isoforms (c) and Thr287 phosphorylated CaMKII (d) in equal protein amount in total homogenate of fatigue resistant (i.e., oxidative) and fatigable (i.e., glycolytic) compartments of the fast* gastrocnemius medialis* muscle and the slow fatigue resistant* soleus* muscle. Lanes originating from different parts of SDS-PAGE gels are separated by black lines. A second band at the height of the *β*M isoform is detected in* soleus* muscle. (e) Graph displays COXIV levels in the oxidative and glycolytic compartments of* gastrocnemius medialis* as determined by western blotting followed by immunodetection. Bars represent mean COXIV levels and symbols represent the levels in individual sample pairs. Symbols being connected by a stippled line reflect intra-animal pairs. The* P*-level of the difference between red and white GM is indicated (paired *t*-test). (f) Example immunoblot showing the detection of COXIV protein and the actin loading control in the oxidative and glycolytic compartments of* gastrocnemius medialis*.

Four CaMKII isoforms, that is, *β*M, *δ*A, and *δ*d/*γ*B were identified in rat* gastrocnemius* muscle in accordance with Rose [12] and Bayer ([13]; Figures 2(a) and 2(b)).* In vitro* autokinase assays confirmed the specificity of detecting CaMKII phosphorylation at Thr287.

The abundance of CaMKII isoforms did not differ between the glycolytic and oxidative compartment of* gastrocnemius medialis* muscle (Figures 2(c), 2(e)/2(f)). The phosphorylation levels of CaMKII at Thr287 were however 2-fold higher in the glycolytic compared to the oxidative compartment of* gastrocnemius medialis* muscle (*P* ≤ 0.01; Figure 2(d)). In the oxidative muscle,* m. soleus*, lower levels of the *δ*d/*γ*B CaMKII isoform and two bands at the height of *β*M were identified (Figure 2(c)).

### 3.2. CaMKII Phosphorylation in Fast-Type Muscle* In Silico* is Graded by the Content of Mitochondria

**Figure 3:**
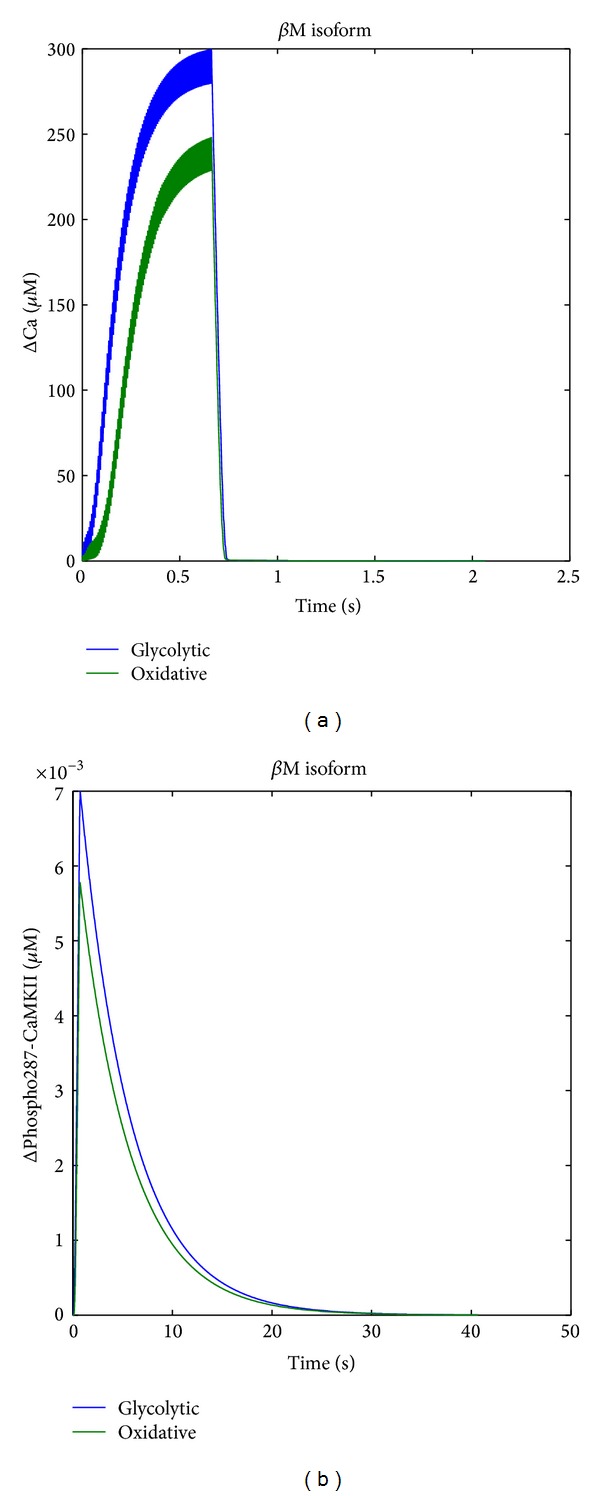
Thr287 phosphorylation of CaMKII in fast muscle compartment after paced contractions* in silico*. (a)(b) Line graphs displaying the level changes of calcium (a) and CaMKII-Thr287 (b) in the glycolytic and oxidative compartments of* gastrocnemius* muscle after an input signal of 100 pulses at 150 Hz.

Calculations were run using CaMuZclE to assess CaMKII-Thr287 phosphorylation in the two compartments of the fast-type* gastrocnemius medialis* muscle after pacing the muscle with a fast-type, 150 Hz protocol. The results of the calculations for these two* gastrocnemius* compartments are shown in Figure 3.

The* in silico* experiment demonstrated a further pronounced increase in Thr287 phosphorylation of CaMKII in the glycolytic compared to the oxidative compartment of the* gastrocnemius medialis* muscle after paced contractions. Essentially, the same result arose when the calculations were run for individual CaMKII isoforms, yet the degree of peak Thr287 phosphorylation differed in the order *β*M > *δ*d/*γ*B > *δ*A (data not shown).

**Figure 4:**
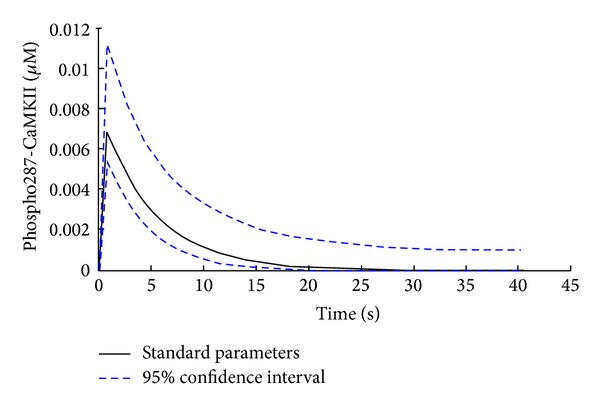
Phospho-Thr287-transient of *β*M CaMKII with 95% confidence intervals. Poisson distribution fitted to the set of output transients obtained by 4000 Latin hypercube-sampled input parameters sets from 75% to 125% of default values. The stimulation protocol with a pulse train of 100 pulses at 150 Hz was used and the model was set up for fast glycolytic muscle. The black line indicates the output transient using the default parameter set. The stippled blue lines indicate the upper and lower 2.5% confidence limit of the Poisson distribution.

Multiparametric sensitivity analysis was carried out to assess the influence of the model parameters on the simulation of calcium-induced Thr287 phosphorylation of CaMKII (Figure 4). Table 1 shows these results in order of decreasing influence of the assessed parameters.

### 3.3. CaMKII Phosphorylation after Paced Contractions in Glycolytic and Oxidative Compartment of Fast-Type Muscle* In Situ*

**Figure 5:**
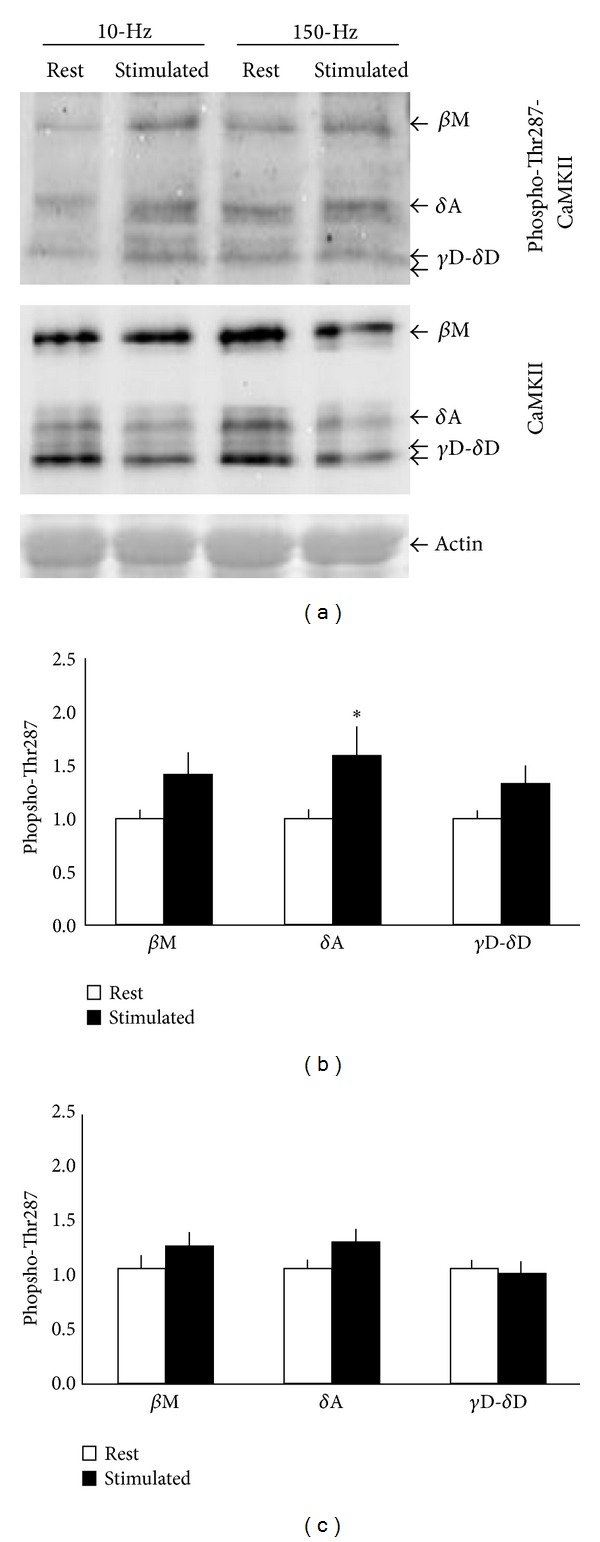
Thr287 phosphorylation of CaMKII in glycolytic and oxidative fast muscle compartment after paced contractions* in situ*. (a) Immunoblot showing the phospho-Thr287-CaMKII signal in stimulated and resting control* gastrocnemius medialis* muscle (oxidative compartment) after stimulation of the sciatic nerve with a train of 100 pulses of a slow (i.e., 10 Hz) or a fast (i.e., 150 Hz) motor unit-targeted protocol [9]. (b)(c) Bar graphs visualizing the mean + SE of changes in phospho-Thr287-CaMKII levels with stimulation of the oxidative (b) and glycolytic (c) compartment of* gastrocnemius medialis.* Data reflect combined values from the stimulation 10-Hz and 150-Hz protocol. ∗denotes *P* < 0.05 versus rest (two-sided paired *t*-test, *n* = 12–14).

Contraction of* gastrocnemius medialis *muscle was stimulated by applying electric pulses at 10 Hz or 150 Hz to the sciatic nerve (Protocols 1 and 2). The biochemical characterisation identified an increase in Thr287-phosphorylation of all combined CaMKII isoforms in the oxidative compartment of* gastrocnemius medialis *muscle (*P* = 0.002). The analysis of individual isoforms demonstrated increased phospho-Thr287 levels of *δ*A CaMKII (*P* = 0.02), but not *β*M (*P* = 0.07) and *δ*D/*γ*B CaMKII (*P* = 0.17), in the oxidative muscle compartment (Figures 5(a) and 5(b)). The levels of neither CaMKII isoform differed between resting and 150 Hz stimulated in the oxidative compartment (all *P*-values > 0.30; data not shown).

In the glycolytic compartment, Thr287 phosphorylation of CaMKII tended to be less affected than in the oxidative portion of* gastrocnemius medialis* after stimulation (*P* = 0.055, for the interaction of “stimulation” × “muscle compartment”). In the glycolytic portion none of the CaMKII isoforms demonstrated regulation after stimulation (Figure 5(c)). No interaction effect was identified between stimulation with the slow versus the fast-type protocol in either portion of* gastrocnemius medialis *muscle for Thr287 phosphorylation.

### 3.4. Frequency Dependent *δ*A CaMKII Isoform Phosphorylation in the Slow Oxidative Compartment

**Figure 6:**
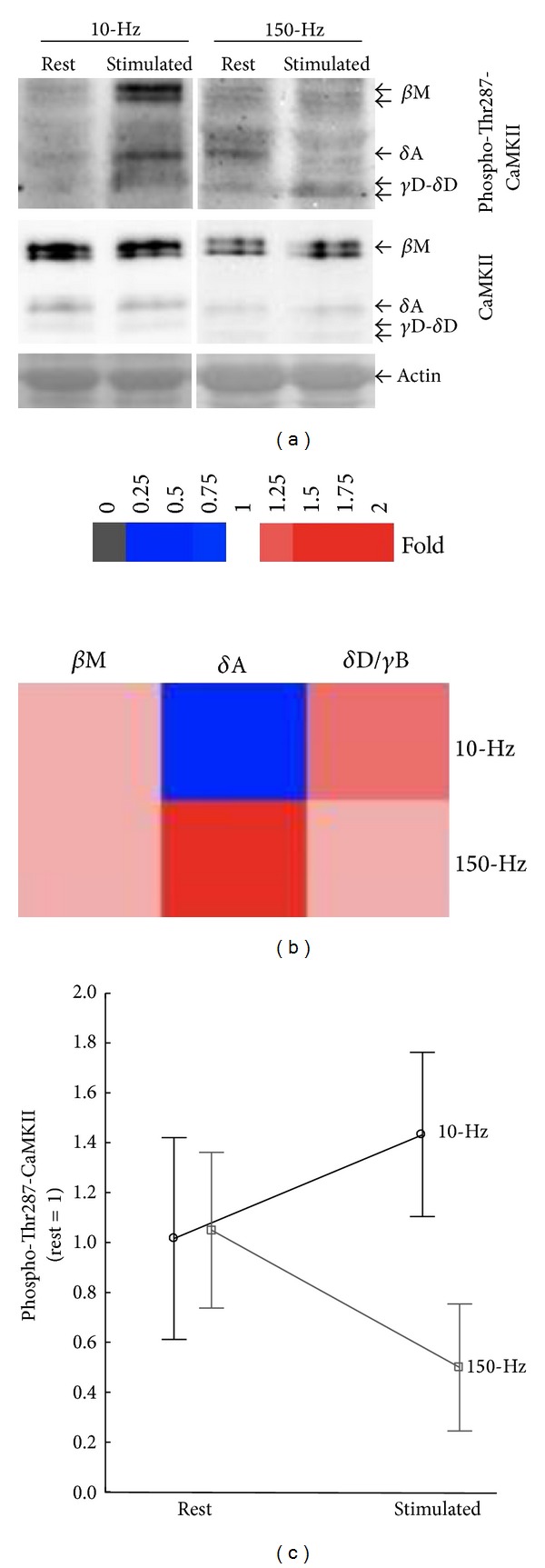
Frequency dependent CaMKII isoform autophosphorylation in slow oxidative muscle after paced contractions* in situ*. (a) Example of Thr 287 phosphorylation of CaMKII isoforms in the stimulated (stim)* soleus* muscle and its contralateral control (rest) after electric stimulation with 150 Hz (Protocol 1) and 10 Hz (Protocol 2). The position of the respective bands is indicated by an arrow. Please note the presence of a second band at the height of the *β*M isoform in stimulated muscle. (b) Composite figure visualising the fold changes in CaMKII-Thr287 content of CaMKII isoforms for slow and fast motor unit-targeted stimulation for CaMKII isoforms in slow oxidative* soleus* muscle in a colour code. (c) Line graph resolving the interaction effect of stimulation frequency on Thr287 phosphorylation of the *δ*A-CaMKII isoform. Repeated ANOVA with post hoc test of Fisher, *n* = 6–10.

We assessed CaMKII isoform phosphorylation in a third muscle of the* triceps surae* complex with a slow-oxidative phenotype, that is, the* m. soleus*, after stimulation with the slow-type (10 Hz) and fast-type (150 Hz) protocol, respectively (Figure 6(a)). The characterisation identified a trend for an interaction between CaMKII isoform and stimulation frequency (*P* = 0.08). When assessing phosphorylation separately per CaMKII isoform we identify a frequency-dependent Thr287 phosphorylation of the *δ*A-CaMKII isoform (*P* = 0.017; Figure 6(b)). Phospho-Thr287 levels of this isoform were selectively downregulated after electric stimulation with the fast-type protocol, but it was increased with the slow-type protocol (Figure 6(c)).

### 3.5. Muscle Specific CaMKII Activation by Repeated Tetanic Contractions of Gastrocnemius Muscle

**Figure 7:**
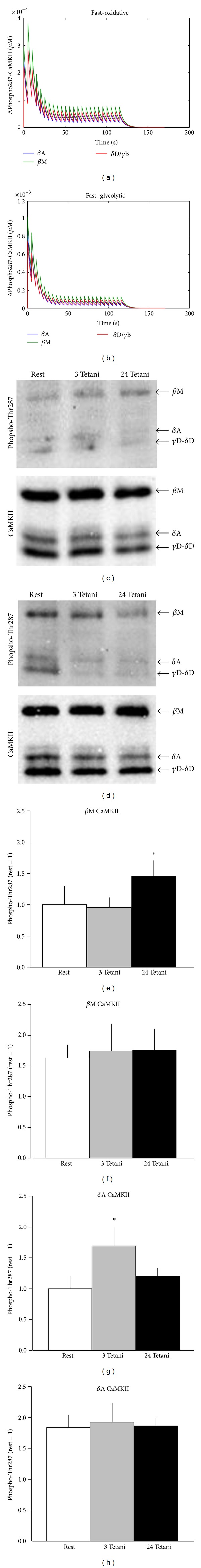
Isoform specific CaMKII autophosphorylation with repeated contractions* in situ*. (a)(b) Line graph of the calculated changes in Thr287 phosphorylation of CaMKII isoforms in the oxidative (a) and glycolytic (b) compartment of* gastrocnemius medialis* muscle as a function of the number of tetanic contractions as paced with repeated trains of 30 pulses of 150 Hz with 4.8 seconds of rest between trains. (c)–(h) Representative immunoblots (c)(d) and bar graphs of mean (e)–(h) of Thr287 phosphorylated CaMKII isoforms in the oxidative (c) (e) (g) and glycolytic (d) (f) (h) compartment of* gastrocnemius medialis* muscle after 3 and 24 repeated tetanic contractions stimulated via the sciatic nerve with trains of 30 pulses at 150 Hz. ∗denotes *P* < 0.05 versus rest (ANOVA with post hoc test of Fisher, *n* = 6).

We investigated whether CaMKII activation in glycolytic and oxidative compartment of fast-twitch muscle would be affected with further repetitions of tetanic contractions. The computational model predicted a rapid increase in CaMKII phosphorylation for all isoforms after repeated tetanic contractions in the oxidative portion of the* gastrocnemius* muscle which followed the order *β*M > *δ*D/*γ*B > *δ*A. Thr287 phosphorylation had early maxima after 1-2 contractions before falling to near resting values (Figures 7(a) and 7(b)).

Except for the *δ*a isoform, the simulated biphasic relationship between phospho-Thr287 and the number of contraction was not what we found experimentally* in situ*. There was a significant interaction effect of the stimulation and compartment (*P* = 0.02) for Thr287 phosphorylation of CaMKII* in situ*. Experiments* in situ* reproduced the model observations on the sensitivity of phospho-Thr287 content of CaMKII in the oxidative compartment of* gastrocnemius medialis* and pointed out isoform-specific phosphorylation in this muscle. Thr287 phosphorylation of the *δ*A CaMKII isoform remained increased after three tetanic contractions but ceased after 24 contractions (Figure 7(g)). By contrast Thr287 content of the *β*M isoform was first significantly increased after 24 tetanic contractions (Figure 7(e)).

## 4. Discussion 

The contribution of muscle contractions to activities of daily living varies from single short duration, for the purpose of acceleration, to continuous contractions being repeated for minutes to hours at a given load until fatigue occurs. While the calcium-induced regulation of myocellular ATPases that produce contractile output is relatively well understood [32], knowledge of calcium-induced regulation of phosphotransferase activities is limited [7, 14]. Towards this end we established a new computational model, CaMuZclE, to investigate whether changes in Thr287 phosphorylation of the calcium-receptive phosphotransferase, CaMKII, following electrically paced contractions could be predicted. CaMKII has been shown to encode information provided by the frequency and amplitude of calcium transients [24] and to regulate excitation-contraction and excitation-transcription coupling [33, 34]. It is unclear which CaMKII isoforms mediate these effects in skeletal muscle but their autophosphorylation pattern in response to fast or slow motor unit-type stimulation might provide clues about CaMKII isoform-specific functions. Therefore, emphasis was put on the elucidation of the response of different isoforms in phenotypically distinct muscles and the influence of slow/fast motor unit stimulation frequencies.

A possible limitation of our approach was that contraction-induced phosphorylation of downstream targets of CaMKII, was not assessed. This experiment is indicated for subsequent studies on CaMKII-regulated signal transduction because Thr287 phosphorylation of CaMKII and* in vitro *measured phosphotransferase activity of CaMKII appears not to be correlated [12]. However, as demonstrated by the same authors, Thr287 phosphorylation of combined CaMKII isoforms increases 1.5-fold in* gastrocnemius* muscle after five repeated tetanic contractions over 10 seconds* in situ *with a further 5-fold increase after 3 minutes of repeated contractions. Our biochemical measurements on the effect of tetanic stimulation over a similar duration (i.e., 0.66 seconds to 2 minutes) are compatible with these results (Figure 5(b)). However, we did not see such large effects on phospho-Thr287 content of CaMKII after one to twenty tetanic contractions and we identify that the *δ*A and *β*M CaMKII isoforms follow a different time course of phosphorylation (Figures 5 and 7). The latter finding indicates a different contribution of CaMKII isoforms to the reported response of combined phospho-Thr287 content of CaMKII with paced contractions* in situ*. The contrasting magnitude in the level increases for the phospho-Thr287 content of CaMKII isoforms respective to Rose [12] is possibly explained by differences in the stimulation protocol and the procedure of harvesting the muscle. In our case, the latter was carried out to separate the three major compartments of the ankle extensor group* triceps surae*. This sampling scheme allowed us to investigate and identify previously unresolved differences in CaMKII phosphorylation between muscle types, that is, the distal (i.e., glycolytic) and proximal (i.e., oxidative)* m. gastrocnemius medialis *and the slow oxidative* m. soleus*. Our biochemical measures identified that phospho-Thr287 content of *δ*A and *β*M CaMKII is increased after externally paced tetanic contractions in the oxidative* gastrocnemius medialis* muscle but is not significantly affected in the glycolytic compartment of the same muscle (Figures 5 and 7). The observed difference in sensitivity of contraction-induced CaMKII phosphorylation between the studied muscle compartments suggests that the anatomical specialisation of muscle fibres is an important factor in the posttranslational regulation of CaMKII.

The role of fibre specialisation for CaMKII isoform autophosphorylation is further corroborated by higher phospho-Thr287 levels of CaMKII at rest between the oxidative and glycolytic portion of* gastrocnemius medialis* (Figure 2). The mechanism behind this baseline difference awaits further exploration. Interestingly, however, the fast-fatigable white* vastus lateralis* muscle of rats responds to repeated voluntary running with an increase in autonomous CaMKII activity [18]. Autonomous phosphotransfer activity of CaMKII reflects increased Thr287 phosphorylation and a subsequent conformation change in the CaMKII enzyme [15]. This suggests a possible contribution of fibre recruitment during cage activity to the baseline differences in phospho287 content of CaMKII between the oxidative and glycolytic compartment of* gastrocnemius medialis* muscle.

Our investigation demonstrates that autophosphorylation of CaMKII isoforms in skeletal muscle depends on the duration of muscle activity. Interestingly, phospho-Thr287 content of the *δ*A isoform was transiently increased and faded after twenty-four tetanic contractions of* gastrocnemius medialis *muscle, when phosphorylation of the major CaMKII isoform of skeletal muscle, *β*M, increased (Figures 7(e)/7(g)). The findings suggest that the *β*M CaMKII isoform of skeletal muscle does, like the *α*CaMKII isoform in neurons, act as a decoder of neuronal input [25]. *β*M CaMKII regulates calcium release from the sarcoplasmic reticulum [7, 14] whereas the *δ*A CaMKII isoform has been demonstrated to affect nuclear processes and modify cardiac growth [22, 23]. Collectively the findings highlight a possible contribution of isoform-specific CaMKII autophosphorylation to excitation-induced signalling in skeletal muscle.

The observed Thr287 phosphorylation of *β*M CaMKII* in situ* (Figure 2), and its association with muscle fatigue (as indicated by a 30% decrease in maximal tetanic force), is in line with the proposition by Tavi [14] on a summation of CaMKII autophosphorylation with repeated contractions. The latter observation was however not diversified for CaMKII isoforms and verified with biochemical analysis. The mechanistic basis of this effect is therefore unclear. Our report points out that autophosphorylation of the *β*M isoform rises slower than *δ*A CaMKII isoform after repeated contractions* in situ* (Figures 7(e)/7(g)). This is intriguing because *β*M localizes to the sarcoplasmic reticulum from which intracellular calcium is released [13] and because sensitivity analysis identifies that the most influential parameter for the rate of CaMKII autophosphorylation, kbta [20], is considerably higher for the *β*M than the *δ*A CaMKII isoform (Table 1).

By contrast, the calmodulin affinity rate constants, which are derived from the parameter kbi, are of lesser importance for our model (0.06 versus 0.32, Table 1), indicating that instead of the calmodulin affinity, the rate constant of autophosphorylation is the rate-determining step. At higher values of this rate constant of autophosphorylation, however, the calmodulin affinity to CaMKII becomes a more important parameter as binding to calmodulin needs to occur before CaMKII autophosphorylation can take place [15]. Both parameters need to be elevated to facilitate a higher total rate of autophosphorylation. Accordingly, this influence is specifically more pronounced for the *β*M isoform due to its higher rate constant [20]. We therefore speculate that the* in situ* observed pattern of *β*M CaMKII isoform autophosphorylation (Figures 7(e)/7(g)) reflects differences in the threshold of calmodulin-dependent autophosphorylation for CaMKII isoforms, or calmodulin availability, rather than kinetic constants of autophosphorylation as characterized* in vitro* [20, 24].

A major finding in this regard was that Thr287 phosphorylation of the *δ*A CaMKII isoform is highly sensitive to excitation and differentiates the response of the fatigue-resistant* soleus* muscle between slow and fast motor unit–type stimulation (Figures  5 and 6; [1]). Interestingly, the increased phospho-Thr287 content of *δ*A CaMKII was only observed with the stimulation frequency corresponding to the “natural” recruitment frequency of the motoneuron in this muscle, that is, 10 Hz; [1, 8]. This supports the idea that elevated *δ*A CaMKII Thr287 phosphorylation in oxidative muscle types depends on the recruitment pattern of motor units. As the* soleus* muscle is also stimulated by the 150 Hz protocol our findings bear the notion that it is the high frequency tetanic excitation that leads to a dephosphorylation of CaMKII *δ*A.

In the course of our investigation, we have established a new computational model that connects calcium dynamics to downstream activation of the regulator of excitation-contraction coupling, CaMKII, in muscle fibers. We used this model to predict possible CaMKII isoform differences in level alterations of Thr287 phosphorylation between stimulation protocols and motor unit types. The phosphatase concentration* in silico* was calibrated by taking the measured decay of Thr287 phosphorylation of CaMKII into account. Of interest in this regard is the fact that* in situ *we observed only a relatively moderate reduction in the content of CaMKII-Thr 287 phosphorylation during recovery from 24 tetanic contractions (data not shown). These values indicate that Vmax or concentration of the phosphatase activity dephosphorylating CaMKII is considerably lower than the value used in the first computational model of CaMKII phosphorylation [16], approximating the initially published values [17]. Accordingly the phosphatases PP1 would be far less efficient to dephosphorylate Thr287 on CaMKII in rat skeletal muscle compared to other tissues.

The output of our* in silico* model emphasises that differences in mitochondrial content as seen for the glycolytic and oxidative* gastrocnemius medialis* muscle bring about different alterations in Thr287 phosphorylation of CaMKII with excitation. The model explains this difference in terms of lower increases in free calcium in the sarcoplasma of oxidative muscle fibers due to the calcium buffer capacity of mitochondria [27]. The model also predicted that Thr287 phosphorylation of CaMKII would fall after an initial peak fade with repeated contraction (Figure 3), a prediction that was confirmed by the observed transient increase in phospho-Thr287 content of *δ*A CaMKII (Figure 7(g)). Our approach can serve as an example as to how* in silico* data can be used to plan experiments and interpret biological data.

However, the results from our modelling did not predict the* in situ* observed differences in the time course of Thr287 phosphorylation between CaMKII isoforms and the discrete influence of the muscle phenotype. For instance modelling predicted a similar transient increase in phospho-Thr287 content of *β*M and *δ*A CaMKII after tetanic contractions but which differed from the data gathered* in situ *(Figure 3 versus Figures 7(a)/7(b)). As well, the predictions on the larger increase in Thr287-phosphorylation in the glycolytic relative to the oxidative compartment of* m. gastrocnemius medialis* differed from the measured data (Figure 3). This disconnection between* in silico *and* in situ *data emphasises that further experimental input from measured parameters, specifically those addressing influential model parameters (Table 1) and their relationship to baseline values and threshold of calcium-induced activation, and *β*M and *δ*A isoform localization, is necessary to refine the model.

## 5. Conclusions

Our findings provide the first evidence that CaMKII isoforms serve as muscle and frequency-specific sensors of muscle excitation. This indicates that aside from the characteristics of a contractile protocol, isoform and muscle type-specific activation of CaMKII must be taken into consideration when interpreting the physiological activation of reactions downstream of CaMKII with muscle contraction* in vivo*. Results from our computational investigations offer a first consolidation of the experimental observations and indicate that the boundary conditions for the modelling of CaMKII regulation need to be adjusted to reveal a mechanistic explanation of CaMKII autophosphorylation in function of calcium dynamics. 

## Figures and Tables

**Table 1:** Relative influences of the model input parameters on the transient in phosphoThr287-CaMKII.

Parameter	Relative influence
kbta	0.32
kPP1	0.24
CaMax	0.22
KmPP1	0.20
CaPump	0.11
kbi	0.06
kbtc	0.06
kTrop1	0.04
kMitCa2	0.04
kDye2	0.03
kMitCa1	0.03
kATP2	0.03
kCaM42	0.03
kParvCa1	0.03
kParvCa2	0.03
kMitMg1	0.03
kDye1	0.03
kATP1	0.03
kbtb	0.03
kCSQN1	0.02
kCaM0Boff	0.02
KdPump	0.02
kMitMg2	0.02
kCaM20	0.02
kTrop2	0.02
kCSQN2	0.02
kParvMg1	0.02
kParvMg2	0.02

Values were calculated as described under multiparametric sensitivity analysis in Section 2.6 “Model Development” of the “Materials and Methods” section. A higher value indicates a larger influence. The most influential parameters are the speed of auto- and dephosphorylation (i.e, kbta, kPPi) and the activity of the calcium pumps (i.e, CaMax, CaPump). For abbreviations consult Tables 2 and 3.

**Table 2:** Initial concentrations.

Name	Value	Unit	Description	Reference
CaMKIItot	1	[*μ*M]	Total CaMKII concentration	http://dare.ubvu.vu.nl/handle/1871/40249
CaMfreeM	0.15	[*μ*M]	Total CaM concentration	http://dare.ubvu.vu.nl/handle/1871/40249
BfreeM	20	[*μ*M]	Total CaM buffer concentration	Saucerman [16]
PP1tot	0.15	[*μ*M]	PP1 concentration	Saucerman [16]
Mg	1000	[*μ*M]	Magnesium concentration sarcoplasm	Saucerman [16]
K	160000	[*μ*M]	Potassium concentration sarcoplasm	Saucerman [16]
CaSRfree	1000	[*μ*M]	Initial calcium concentration in sarcoplasmic reticulum	http://www.tue.nl/en/publication/ep/p/d/ep-uid/247565/
CSQNT	60000	[*μ*M]	Total calsequestrin concentration	http://www.tue.nl/en/publication/ep/p/d/ep-uid/247565/
Ca	0.1	[*μ*M]	Initial calcium concentration sarcoplasm	http://www.tue.nl/en/publication/ep/p/d/ep-uid/247565/
TropT	205	[*μ*M]	Total troponin C concentration	http://www.tue.nl/en/publication/ep/p/d/ep-uid/247565/
ParvT	1500	[*μ*M]	Total parvalbumin concentration	http://www.tue.nl/en/publication/ep/p/d/ep-uid/247565/
MitT	100	[*μ*M]	Total mitochondria concentration	[35]
ATPT	8000	[*μ*M]	Total APT concentration	http://www.tue.nl/en/publication/ep/p/d/ep-uid/247565/
DyeT	81	[*μ*M]	Total dye concentration	http://www.tue.nl/en/publication/ep/p/d/ep-uid/247565/
Pb2R	0	[*μ*M]	Initial CaMKII Pb2 in state	http://www.tue.nl/en/publication/ep/p/d/ep-uid/247565/
PbR	0	[*μ*M]	Initial CaMKII Pb in state	http://www.tue.nl/en/publication/ep/p/d/ep-uid/247565/
PtR	0	[*μ*M]	Initial CaMKII Pt in state	http://www.tue.nl/en/publication/ep/p/d/ep-uid/247565/
Pt2R	0	[*μ*M]	Initial CaMKII Pt2 in state	http://www.tue.nl/en/publication/ep/p/d/ep-uid/247565/
PaR	0	[*μ*M]	Initial CaMKII PaR in state	http://www.tue.nl/en/publication/ep/p/d/ep-uid/247565/
PiR	1	[*μ*M]	Initial CaMKII PiR in state	http://www.tue.nl/en/publication/ep/p/d/ep-uid/247565/
XB_*n*_	1	[—]	Initial fraction of total action/myosin chains in XB_*n*_ state	http://www.tue.nl/en/publication/ep/p/d/ep-uid/247565/
XB_*w*_	0	[—]	Initial fraction of total action/myosin chains in XB_*w*_ state	http://www.tue.nl/en/publication/ep/p/d/ep-uid/247565/
XB_*s*_	0	[—]	Initial fraction of total action/myosin chains in XB_*s*_ (force) state	http://www.tue.nl/en/publication/ep/p/d/ep-uid/247565/
XB_*r*_	0	[—]	Initial fraction of total action/myosin chains in XB_*r*_ state	http://www.tue.nl/en/publication/ep/p/d/ep-uid/247565/

**Table 3:** Rate constants.

Name	Value	Unit	Description	Reference
CaMax	25	[*μ*M^−1^]	Ca^2+^ release rate	http://www.tue.nl/en/publication/ep/p/d/ep-uid/247565/
Power1	5	[—]	RyR kinetic parameter	http://www.tue.nl/en/publication/ep/p/d/ep-uid/247565/
tau1	0.609	[s]	RyR kinetic parameter	http://www.tue.nl/en/publication/ep/p/d/ep-uid/247565/
Power2	3	[—]	RyR kinetic parameter	http://www.tue.nl/en/publication/ep/p/d/ep-uid/247565/
tau2	0.774	[s]	RyR kinetic parameter	http://www.tue.nl/en/publication/ep/p/d/ep-uid/247565/
CaPump	20	[*μ*M ∗ ms^−1^]	Maximum SERCA pumping	http://dare.ubvu.vu.nl/handle/1871/40249
KdPump	1	[*μ*M]	Dissociation constant calcium binding to SERCA	http://www.tue.nl/en/publication/ep/p/d/ep-uid/247565/
kAtp1	0.054	[*μ*M^−1^ ∗ ms^−1^]	On rate reaction Ca^2+^-ATP	http://www.tue.nl/en/publication/ep/p/d/ep-uid/247565/
kAtp2	120	[ms^−1^]	Off rate reaction Ca^2+^-ATP	http://www.tue.nl/en/publication/ep/p/d/ep-uid/247565/
kTrop1	0.08	[*μ*M^−1^ ∗ ms^−1^]	On rate reaction Ca^2+^-troponin C	http://www.tue.nl/en/publication/ep/p/d/ep-uid/247565/
kTrop2	0.32	[ms^−1^]	Off rate reaction Ca^2+^-troponin C	http://www.tue.nl/en/publication/ep/p/d/ep-uid/247565/
kParvCa1	0.37	[*μ*M^−1^ ∗ ms^−1^]	On rate reaction Ca^2+^-parvalbumin	http://www.tue.nl/en/publication/ep/p/d/ep-uid/247565/
kParvCa2	0.003	[ms^−1^]	Off rate reaction Ca^2+^-parvalbumin	http://www.tue.nl/en/publication/ep/p/d/ep-uid/247565/
kParvMg1	1.05*E* − 04	[*μ*M^−1^ ∗ ms^−1^]	On rate reaction Mg^2+^-parvalbumin	http://www.tue.nl/en/publication/ep/p/d/ep-uid/247565/
kParvMg2	0.012	[ms^−1^]	Off rate reaction Mg^2+^-parvalbumin	http://www.tue.nl/en/publication/ep/p/d/ep-uid/247565/
kMitCa1	0.37	[*μ*M^−1^ ∗ ms^−1^]	On rate reaction Ca^2+^-mitochondria	http://www.tue.nl/en/publication/ep/p/d/ep-uid/247565/
kMitCa2	0.003	[ms^−1^]	Off rate reaction Ca^2+^-mitochondria	http://www.tue.nl/en/publication/ep/p/d/ep-uid/247565/
kMitMg1	1.05*E* − 04	[*μ*M^−1^ ∗ ms^−1^]	On rate reaction Mg^2+^-mitochondria	http://www.tue.nl/en/publication/ep/p/d/ep-uid/247565/
kMitMg2	0.012	[ms^−1^]	Off rate reaction Mg^2+^-mitochondria	http://www.tue.nl/en/publication/ep/p/d/ep-uid/247565/
kCsqn1	0.1	[*μ*M^−1^ ∗ ms^−1^]	On rate reaction Ca^2+^-calsequestrin	http://www.tue.nl/en/publication/ep/p/d/ep-uid/247565/
kCsqn2	100	[ms^−1^]	Off rate reaction Ca^2+^-calsequestrin	http://www.tue.nl/en/publication/ep/p/d/ep-uid/247565/
kDye1	0.0864	[*μ*M^−1^ ∗ ms^−1^]	On rate reaction Ca^2+^-Dye	http://www.tue.nl/en/publication/ep/p/d/ep-uid/247565/
kDye2	6.05	[ms^−1^]	Off rate reaction Ca^2+^-Dye	http://www.tue.nl/en/publication/ep/p/d/ep-uid/247565/
kCam20	0.01	[ms^−1^]	Ca dissociation from CaM (C-terminal)	Saucerman [16]
kCam42	0.5	[ms^−1^]	2 Ca dissociation from CaM (N-terminal)	Saucerman [16]
kCam0Boff	1.40*E* − 06	[ms^−1^]	CaM dissociation from buffer	Saucerman [16]
kPP1	0.0017	[ms^−1^]	Thr287 dephosphorylation	Saucerman [16]
KmPP1	11	[*µ*M]	Km for Thr287 dephosphorylation	Saucerman [16]
kbi	0.0013	[ms^−1^]	Ca4CaM dissociation from Pb	Gaertner [20]
kbta	0.018	[—]	Polynomial factor autophosphorylation	Gaertner [20]
kbtb	0.015	[—]	Polynomial factor autophosphorylation	Gaertner [20]
kbtc	0.033	[—]	Polynomial factor autophosphorylation	Gaertner [20]
KF50	8.4	[*μ*M]	Dissociation constant Ca-troponin C from XB_*n*_ state	http://www.tue.nl/en/publication/ep/p/d/ep-uid/247565/
NF2	1.68	[—]	Power constant for Ca-troponin C influence on kf1	http://www.tue.nl/en/publication/ep/p/d/ep-uid/247565/
KF502	27	[*μ*M]	Dissociation constant Ca-troponin C from XB_*w*_ state	http://www.tue.nl/en/publication/ep/p/d/ep-uid/247565/
NF2	8	[—]	Power constant for Ca-troponin C influence on kf2	http://www.tue.nl/en/publication/ep/p/d/ep-uid/247565/
kFm1s	0.0045	[ms^−1^]	On rate reaction XB_*n*_-XB_*w*_	http://www.tue.nl/en/publication/ep/p/d/ep-uid/247565/
kFm1	0.045	[ms^−1^]	Off rate reaction XB_*n*_-XB_*w*_	http://www.tue.nl/en/publication/ep/p/d/ep-uid/247565/
kF0	1.00*E* − 03	[ms^−1^]	Rate second reaction XB_*n*_-XB_*w*_	http://www.tue.nl/en/publication/ep/p/d/ep-uid/247565/
kFg1	0.0169	[ms^−1^]	Rate reaction XB_*s*_-XB_*r*_	http://www.tue.nl/en/publication/ep/p/d/ep-uid/247565/
kFg2	0.0337	[ms^−1^]	Rate reaction XB_*r*_-XB_*n*_	http://www.tue.nl/en/publication/ep/p/d/ep-uid/247565/
kFm2	0.13	[ms^−1^]	On rate reaction XB_*w*_-XB_*s*_	http://www.tue.nl/en/publication/ep/p/d/ep-uid/247565/
kFm2s	1	[ms^−1^]	Off rate reaction XB_*w*_-XB_*s*_	http://www.tue.nl/en/publication/ep/p/d/ep-uid/247565/

**Table 4:** CaMKII isoforms.

Name	Value	Unit	Description	Reference
*δ*A				
kbi	0.0013	[ms^−1^]	Ca4CaM dissociation from Pb	Gaertner [20]
kbta	0.018	[—]	Polynomial factor autophosphorylation	Gaertner [20]
kbtb	0.015	[—]	Polynomial factor autophosphorylation	Gaertner [20]
kbtc	0.033	[—]	Polynomial factor autophosphorylation	Gaertner [20]
*β*M				
kbi	0.00054	[ms^−1^]	Ca4CaM dissociation from Pb	Gaertner [20]
kbta	0.043	[—]	Polynomial factor autophosphorylation	Gaertner [20]
kbtb	0.0062	[—]	Polynomial factor autophosphorylation	Gaertner [20]
kbtc	0.019	[—]	Polynomial factor autophosphorylation	Gaertner [20]
*δ*d				
kbi	0.00015	[ms^−1^]	Ca4CaM dissociation from Pb	Gaertner [20]
kbta	0.0053	[—]	Polynomial factor autophosphorylation	Gaertner [20]
kbtb	0.00092	[—]	Polynomial factor autophosphorylation	Gaertner [20]
kbtc	0.066	[—]	Polynomial factor autophosphorylation	Gaertner [20]
*γ*B				
kbi	0.00070	[ms^−1^]	Ca4CaM dissociation from Pb	Gaertner [20]
kbta	0.055	[—]	Polynomial factor autophosphorylation	Gaertner [20]
kbtb	0.0074	[—]	Polynomial factor autophosphorylation	Gaertner [20]
kbtc	0.015	[—]	Polynomial factor autophosphorylation	Gaertner [20]

**Table 5:** Slow glycolytic parameter changes compared to fast glycolytic.

Name	Value	Unit	Description	Reference
Ca	0.15	[*μ*M]	Initial calcium concentration sarcoplasm	http://www.tue.nl/en/publication/ep/p/d/ep-uid/247565/
TropT	102	[*μ*M]	Total troponin C concentration	http://www.tue.nl/en/publication/ep/p/d/ep-uid/247565/
ParvT	0	[*μ*M]	Total parvalbumin concentration	http://www.tue.nl/en/publication/ep/p/d/ep-uid/247565/
MitT	300	[*μ*M]	Total parvalbumin concentration	http://www.tue.nl/en/publication/ep/p/d/ep-uid/247565/
ATPT	5000	[*μ*M]	Total APT concentration	http://www.tue.nl/en/publication/ep/p/d/ep-uid/247565/
CaMax	8	[*μ*M^−1^]	Ca^2+^ release rate	http://www.tue.nl/en/publication/ep/p/d/ep-uid/247565/
Power1	6.71	[—]	RyR kinetic parameter	http://www.tue.nl/en/publication/ep/p/d/ep-uid/247565/
tau1	1.06	[s]	RyR kinetic parameter	http://www.tue.nl/en/publication/ep/p/d/ep-uid/247565/
Power2	2.98	[—]	RyR kinetic parameter	http://www.tue.nl/en/publication/ep/p/d/ep-uid/247565/
tau2	0.98	[s]	RyR kinetic parameter	http://www.tue.nl/en/publication/ep/p/d/ep-uid/247565/
CaPump	1	[*μ*M ∗ ms^−1^]	Maximum SERCA pumping	http://www.tue.nl/en/publication/ep/p/d/ep-uid/247565/
kTrop2	0.16	[ms^−1^]	Off rate reaction Ca^2+^-troponin C	http://www.tue.nl/en/publication/ep/p/d/ep-uid/247565/

**Table 6:** Fast oxidative parameter changes compared to fast glycolytic.

Name	Value	Unit	Description	Reference
MitT	250	[*μ*M]	Total concentration of mitochondria	http://dare.ubvu.vu.nl/handle/1871/40249

## Appendices

### A. Fluxes

#### A.1. Pumps of the Sarcoplasmic Reticulum

The RyRs are modeled using an empirical function, derived from Baylor [36]:

(A.1) Jryr=([Ca]SR−  [Ca]Myo)e−t/τ2 ∗CaMax∗(1−e−t/τ1)power1.

It is based on the opening of the channels inside the RyRs, which are regulated by electrical signals coming from the nerves going via the sarcolemma. Parameters involved are the reference calcium flow, the two characteristic opening times of the channels, and the exponential coefficients describing the weight of the opening times. Furthermore, the flux is made dependent on the difference in calcium concentration to incorporate a diffusion component.

The SERCA pumps are modeled as described in [37]:

(A.2) $$\begin{array}{c} J_{\text{SERCA}}=\frac{\text{CaPump}*{\left[\text{Ca}\right]}_{\text{Myo}}^{2}}{{\left[\text{Ca}\right]}_{\text{Myo}}^{2}+K_{d,\text{pump}}^{2}}. \end{array}$$

The rate is second order, indicating cooperativity, as the pump transports two ions at the same time for one ATP molecule. Other involved parameters are the reference pumping rate and the dissociation constant of calcium binding.

#### A.2. Calcium Buffers

The change in concentration of the buffers calsequestrin, parvalbumin, troponin, ATP, and the mitochondria is modeled using mass-action kinetics. Calsequestrin is a low-affinity, high-capacity buffer inside the SR that stores calcium. Parvalbumin is a high capacity calcium buffer that also binds magnesium, therefore making it a slow onset calcium buffer. Mitochondria also bind calcium and magnesium, with the same parameters as parvalbumin. ATP can buffer calcium as well as magnesium; but using a reduced reaction this can be approximated by only modeling the calcium binding, as changes in total free Mg concentration are small [36]. It is a low-affinity, rapid acting calcium buffer. Calcium binds to troponin to induce contraction. These buffers are modeled using a second order reaction

(A.3) $$\begin{array}{c} J_{\text{buf-Ca}}=k_{\mathrm{on}}*{\left[\text{Ca}\right]}_{\text{Myo}}*{\left[\text{buf}\right]}_{\text{free}}-k_{\mathrm{off}}*{\left[\text{buf}\right]}_{\text{Ca}}, \end{array}$$

with *k*_*on*⁡_ and *k*_*off*⁡_ the rate constants of binding and release of the complex, respectively, for each buffer, [Ca]_Myo_ the calcium concentration in the sarcoplasm, [buf]_free_ the free buffer concentration, and [buf]_Ca_ the concentration of buffer bound to calcium. As parvalbumin also binds to magnesium, a second reaction describes the reaction with magnesium

(A.4) JParv-Mg=kon⁡,Mg∗[Mg]Myo∗[Parv]free −koff⁡,Mg∗[Parv]Mg.

The fluxes describe the change in concentration of the buffers bound to calcium or magnesium. The concentrations of all the other species can then be deduced from these fluxes.

#### A.3. CaM and CaMKII

The CaM and CaMKII reactions are modeled as described by Saucerman [16]. CaM is a calcium sensor that can bind 4 calcium molecules, two to the N-terminal EF hand and two to the C-terminal EF hand of the amino-acid chain. The binding kinetics can be reduced to a three state model to reduce parameters, as binding at each location is cooperative. Furthermore, each state also exists in a buffered form. This model is included in the solver by first calculating the reaction rates, where for each state transition the rate is calculated by taking the binding rate and subtracting the release rate (A.5). These rates are described by mass action kinetics, resulting in a third order reaction as 2 calcium ions bind to one CaM protein at the same time. The reaction rates are then combined into fluxes for each of the six CaM and CaM-buffer states (A.5).

Next, CaMKII reactions are modeled in an equal fashion. CaMKII exists in 6 different states, with 4 of these states together forming the active fraction of CaMKII (Pb, Pt, Pa, Pt2) and 3 of these four states phosphorylated at the threonine 287 site (Pt, Pa, Pt2). Switching between these states is facilitated by either (de) phosphorylation, Ca2CaM binding and release, or Ca4CaM binding and release. Again, the reaction rates are calculated for each state transition (A.5), resulting in either second or third order reactions. Next, the reaction rates are combined into fluxes for each state (A.5). Consider

(A.5) R02=kCaM02∗[Ca]Myo2∗[CaM]−kCaM20∗[Ca2−CaM],R24=kCaM24∗[Ca]Myo2∗[Ca2−CaM] −kCaM42∗[Ca4−CaM],R02B=kCaM02B∗[Ca]Myo2∗[CaM−Buffer] −kCaM20B∗[CaM−Buffer−Ca2],R24B=kCaM24B∗[Ca]Myo2∗[CaM−Buffer−Ca2] −kCaM42B∗[CaM−Buffer−Ca4],R2B=kCaM2Bon∗[Ca2−CaM]∗[Buffer] −kCaM2Boff∗[CaM−Buffer−Ca2],R4B=kCaM4Bon∗[CaM−Buffer]∗[Buffer] −kCaM4Boff∗[CaM−Buffer−Ca4],RCKib=kib∗[Ca4−CaM]∗[Pi]−kbi∗[Pb],RCKib2=kib2∗[Ca2−CaM]∗[Pi]−kb2i∗[Pb2],RCKb2b=kb24∗[Ca]{Myo}2∗[Pb2]−kb42∗[Pb],RCKbt=kbt∗[Pb]−kPP1∗[PP1]∗[Pt]Km,PP1+[CaMKII]tot∗[Pt],RCKtt2=kt42∗[Pt]−kt24∗[Ca]Myo2∗[Pt2],RCKt2b2=kPP1∗[PP1]∗[Pt2]Km,PP1+[CaMKII]tot∗[Pt2],RCKta=kta∗[Pt]−kat∗[Ca4−CaM]∗[Pa],RCKt2a=kt2a∗[Pt2]−kat2∗[Ca2−CaM]∗[Pa],RCKai=kPP1∗[PP1]∗[Pa]Km,PP1+[CaMKII]tot∗[Pa],JCaM=−R02−R0B,JCa2-CaM=R02−R24−R2B+RCKt2a−RCKib2,JCa4-CaM=R24−R4B+RCKta−RCKib,JCaM-Buffer=R0B−R02B,JCaM-Buffer-Ca2=R02B−R24B+R2B,JCaM-Buffer-Ca4=R24B+R4B,JPi=−RCKib−RCKib2+RCKai,JPb=RCKib+RCKb2b−RCKbt,JPb2=RCKib2−RCKb2b+RCKt2b2,JPt=RCKbt−RCKtt2−RCKta,JPt2=RCKtt2−RCKt2b2−RCKt2a,JPa=−RCKai+RCKta+RCKt2a.

Some of the rate constants depend on the concentration of the ions magnesium and potassium. Phosphorylation is caused by the protein phosphorylating itself (termed autophosphorylation), where dephosphorylation is facilitated by the protein PP1.

#### A.4. Force

The force is modeled as described by Groenendaal [29], which compared different models for coupling the troponin concentration to force production by the myofibrils. It is described by a four state model of the actin and tropomyosin chains. Calcium bound troponin C (ca-TropC) facilitates the transformation between states. Without ca-TropC all the myosin chains are in the XB_*n*_ state. When ca-TropC is formed, the myosin chains go through a series of states called the cross bridge cycle, thereby inducing contraction of the muscle fibers and force production.

The cycle is modeled using rate constants, with some dependent on the concentration of ca-TropC:

(A.6) JXBn=−k1([Trop−Ca])∗XBn−kF,0∗XBn+kF,−1∗XBw+kFg1∗XBr,JXBw=k1([Trop−Ca])∗XBn+kF,0∗XBn −kF,−1∗XBw−k2([Trop−Ca])∗XBw+kF,−2∗XBs,JXBs=k2([Trop−Ca])∗XBw−kF,−2∗XBs−kF,g1∗XBs,JXBr=−k(F,g2)XBr+kF,g1∗XBs,kF,−1∗([Trop−Ca]KF,50)NF =k1([Trop−Ca]),kF,−2∗([Trop−Ca]KF,502)NF2 =k2([Trop−Ca]).

The force is described by the fraction of myosin chains in the XB_*s*_ state. This model is not able to produce an actual quantitative measure of force, as this is beyond the scope of this research. The force output of this model is therefore normalized between zero and one to compare it with experimental data.

#### A.5. Contractile Phenotypes and CaMKII Isoforms

The model is then further expanded to describe the different CaMKII isoforms *β*M, *δ*A, *δ*d, and *γ*B. Although the functions of the different isoforms are generally equal, they reside in different compartments in the cell and have a different structure and molecular weight, resulting in different reactions constants.

Furthermore, three different twitch types of muscle fibers are modeled, fast glycolytic, slow glycolytic, and fast oxidative. Compared to fast glycolytic, slow glycolytic fibers have less active RyRs and less active SERCA pumps, resulting in a lower baseline of calcium concentration in the sarcoplasm. Furthermore, the fibers have a lower concentration of the calcium buffers ATP and parvalbumin. Troponin C has a lower amount of binding sites, which is modeled as a lower concentration. Fast oxidative uses the same parameters as fast glycolytic but has a higher amount of mitochondria compared to the other fiber types.

#### A.6. Equilibration

Before the model can be run the total amounts of all derivative species need to be calculated; as for most species only the total amount is known. If the amount of free calcium and free buffer inside the sarcoplasm in equilibrium is known, the concentrations of calcium bound to buffer can be calculated by setting the fluxes of calcium binding and release equal to each other (A.7). If only the total amount of buffer is known, the Michaelis-Menten equilibrium approximation can be used (A.7). For the competitive binding of calcium and magnesium to parvalbumin or mitochondria (A.7) can be used:

(A.7) [buf]Ca∗k2=[buf]free∗[Ca]Myo∗k1,[buf]Ca=[buf]free∗[Ca]Myo∗k1k2,[buf]Ca=[buf]free∗[Ca]Myo(k1/k2)+[Ca]Myo,[buf]tot=[buf]free+[buf]Ca+[buf]Mg,[buf]tot=[buf]free+[buf]free∗[Ca]Myok2/k1 +[buf]free∗[Mg]k2/k1,[buf]tot=[buf]free∗(1+[Ca]Myok2/k1+[Mg]k2/k1),[buf]free=[buf]tot1+([Ca]Myo/(k2/k1))+([Mg]/(k2/k1)).

### B. Model Parameters

For more details see Tables 2, 3, 4, 5, and 6.

## Supplementary Data

Program lines and source codes will be made available upon request.
